# The Effect of Two Acute Bouts of Exercise on Oxidative Stress, Hematological, and Biochemical Parameters, and Rectal Temperature in Trained Canicross Dogs

**DOI:** 10.3389/fvets.2022.767482

**Published:** 2022-03-11

**Authors:** Vladimira Erjavec, Tomaž Vovk, Alenka Nemec Svete

**Affiliations:** ^1^Small Animal Clinic, Veterinary Faculty, University of Ljubljana, Ljubljana, Slovenia; ^2^The Chair of Biopharmaceutics and Pharmacokinetics, Faculty of Pharmacy, University of Ljubljana, Ljubljana, Slovenia

**Keywords:** dogs, canicross, exercise-induced oxidative stress, antioxidant enzymes, lipid peroxidation, hematology, biochemistry, body temperature

## Abstract

Canicross is a sport discipline that connects human and canine athletes in running. Changes in physiological, hematological, and biochemical parameters, and exercise-induced oxidative stress have not been thoroughly characterized in canicross dogs. The aim of our study was the assessment of the health status of trained canicross dogs that were subjected to two acute bouts of exercise with their owners during the training season. Health status was assessed by measuring the rectal temperature, hematological and biochemical parameters, as well as blood oxidative stress parameters (plasma malondialdehyde, lipid peroxidation marker; whole blood glutathione peroxidase and erythrocyte superoxide dismutase1, antioxidant enzymes) before and during a two-day canicross training session and after a 24-h rest period. Seven trained canicross dogs (three females/four males) aged 12–120 months were included in the study. Blood samples were collected before and immediately after the first acute bout of exercise (day 1), after the second acute bout of exercise (day 2), and after 24 h of rest (day 3). Rectal temperature was measured at the same time as blood sample collection. The majority of hematological and biochemical parameters remained within reference ranges at all sampling times. Rectal temperature was significantly higher after training on days 1 and 2 compared to resting temperature on day 3. Hematological parameters did not change significantly; however, there were significant differences in urea, creatinine, creatine kinase, and triglycerides between specific sampling times. Despite significant changes, these biochemical parameters remained within reference ranges. Significant changes in biochemical parameters seem to reflect the dogs' physiological response to each acute bout of exercise, considering all biochemical parameters and rectal temperature returned to pre-exercise values after a 24-h rest period (day 3). No significant differences in oxidative stress parameters were found between any sampling times. Relatively high erythrocyte superoxide dismutase1 activity at all sampling times may indicate that the canicross dogs are adapted to training by an increased expression of antioxidant enzymes. Based on our results, we can conclude that the trained canicross dogs included in our study were healthy, in good physical condition, and fit for the two acute bouts of field exercise.

## Introduction

Canicross is a sport that can promote human and canine athletes in running. Typically, the dogs are connected to the handler, the human runner, by a shock-absorbing leash such as a bungee cord or elastic line connected to a pull harness on the dog and a waist belt on the human (1). It was originally popular as off-season training for sled dogs, but has since become a popular stand-alone sport. In the early stages of the sport, sled dogs such as the Siberian Husky or Alaskan Malamute were typically used. Today, the sport is open to all breeds, both large and small; however, toy and flat-faced breeds are not suitable partners for long-distance races (2).

The distance of canicross races ranges from relatively short “sprint” distances of approximately 5 km to longer competitive distances of 45 km or more (3). The canicross runners usually compete with their dogs over two consecutive days with identical courses on both days, while Greyhound races are usually held once per week (4, 5) and the sled-dog races for up to 14 days to be completed (6). The canicross is the sport that can promote increased physical activity and encourage people to exercise with their dog to improve race times, competitive performance, health and fitness in both species (7). Encouraging owners to promote increased physical activity could prove to be dangerous for dogs running long distances, which may predispose these dogs to potential risk of physical exertion. Heat-related illness could be an issue in the summer months with higher ambient temperatures and humidity (7–10). These will become an ever-greater threat as climate change causes global temperatures to rise (8). Thus, establishing reference rectal temperature responses and associated clinical characteristics at lower ambient temperatures is clinically relevant and important.

In order to provide useful information on physical fitness of athlete dogs, it is important to identify physiological parameters for rapid clinical evaluation. Physical fitness in athlete dogs has been defined as “the body's ability to maintain internal physiological balance as close as possible to the state of rest during physical exertion and to promptly restore the altered balance after exercise” (11, 12). A marked increase in body temperature, heart rate, and blood lactate concentration after exercise, as well as alterations in hematological and biochemical parameters, have been reported not only in human athletes (13–21), but also in dogs subjected to various forms and/or intensities of exercise (22–41). With the exception of rectal temperature (7), such results have not been reported in canicross dogs.

Physical activity is associated not only with changes in physiological, hematological, and biochemical parameters, but also with exercise-induced oxidative stress. During exercise, there is increased production of reactive oxygen species (ROS), as well as increased consumption of antioxidants, which can lead to exercise-induced oxidative stress causing oxidative damage to lipids, proteins and DNA (42–44). In addition, exercise-induced oxidative stress is thought to contribute to muscle fatigue and muscle fibre damage, which can lead to exercise intolerance and poor performance (43, 45, 46). While high concentrations of ROS may be harmful, lower/physiological concentrations of ROS play an important role in normal cellular functions, such as cell signaling and regulation of gene expression. In addition, moderate concentrations of ROS are thought to be necessary for muscle adaptation to exercise training by modulating muscle contraction and upregulation of antioxidant genes, thus contributing to resistance to oxidative damage (44, 47–51). Exercise-induced oxidative stress has been extensively studied in human (48, 50, 52) and equine athletes (53–56), but much less so in canine athletes. Changes in various oxidative stress parameters following exercise have been reported in sled dogs (57–62), in dogs during agility exercises (34), in hunting dogs (35, 63), search and rescue dogs (64) and in therapy dogs (65), but not in canicross dogs undergoing regular acute bouts of exercise training session.

Despite published reports investigating the effects of different types of physical activity on physiological, hematological, biochemical, and oxidative stress parameters in canine athletes, the results cannot be easily extrapolated to canicross dogs. This is because of the unique characteristics of the sport of canicross, which include the relatively longer duration of high intensity exercise that canicross dogs experience, along with the additional load of pulling against a human athlete that limits the dog's speed. Due to rapidly growing popularity of this sport increasing numbers of novice and unfit animals may be presented to canicross races. Most dogs competing in canicross are owned by pet owners, with limited prior experience of training and preparing canine athletes. On the other side, most sled dogs, Greyhounds and military working dogs are under the influence of experienced canine athlete trainers, strict policies and protocols regarding veterinary checks prior to racing, and restrictions on acceptable race conditions (4–6, 37, 66). Furthermore, canicross races are not comparable to Greyhound and sled-dog races. While Greyhound races are characterized by the short distance, short duration, and single breed involvement, sled-dog racing is also breed specific (Samoyed, Alaskan Malamute, Siberian Husky) and covers very long distances ranging from 500 to 1,569 km in a continuous competition lasting for up to 14 days to be completed and takes place under extremely challenging environmental conditions (the temperature is below 0°C and the snow conditions) (6). Canicross sport is placed between these two extremes and introduces the added resistance of pulling against the human athlete, the two-day format of most races, and far more variable breed involvement. In addition, physical exertion has been shown to be the most common cause of heat-related illness in dogs in the United Kingdom and Israel (8, 10). The aim of our study was assessment of the health status of trained canicross dogs that underwent regular two acute bouts of exercise during two consecutive days with their owners during the training season. Health status was assessed by measuring the rectal temperature, hematological and biochemical parameters, as well as oxidative stress parameters, plasma malondialdehyde (MDA), lipid peroxidation marker; whole blood glutathione peroxidase (GPX) and erythrocyte superoxide dismutase1 (erythrocyte SOD1), antioxidant enzymes, before and during the two-day exercise sessions and after a 24-h rest period.

## Materials and Methods

### Canine Characteristics and Training History

Seven clinically healthy, client-owned, trained canicross dogs were included in the study. All dogs were kept as companion animals at their owners' homes. They were in active training at the time of study entry, and owners had not observed significant changes in physical performance during the few months preceding the study. The dogs exercise regularly for two consecutive days (20–30 min per day) followed by a rest day for at least 4 months before this study. All dogs, except one had previously competed in canicross races, so the current season was not their first racing season.

### Acute Exercise Protocol

As on every training day, on both exercise days each dog was transported by the owner's car to the location where the two acute bouts of exercise were performed. After the 20-min acclimatization period, rectal temperature was measured with a digital thermometer and pre-exercise blood samples were collected. None of the dogs had been exercised for 48 h before the first blood sampling. Exercise was always performed in the late afternoon and at least 6 h after feeding to prevent possible gastric volvulus and the effect of feeding on blood parameters. The dogs were allowed small amount of water 30 min before exercise and were not allowed to drink during the 30 min before exercise and during the first 30 min of recovery. The normal owner's practice is to withhold the water to prevent vomiting. The dog harness was attached to the owner with the leash on which the dog was actively pulling. The owners ran with their dogs for two consecutive days over a distance of 4.5 km, followed by a rest day. The terrain was flat to moderately hilly and was one of the terrains in which dogs usually exercise. The time in which the exercise was completed was used to calculate the average running speed, which ranged from 12 to 22 km/h with an average speed of 15 km/h.

The ambient temperature ranged from 18.7 to 19.8°C and from 12.2 to 13.5°C on day 1 and day 2, respectively. Humidity ranged from 28 to 31% and from 64 to 77% on day 1 and day 2, respectively. Universal thermal climate index (UTCI) values ranged from 18 to 19.1°C on day 1 and from 13.6 to 15.2°C on day 2. There was no wind on the first and second day of the exercise. On day 2, the amount of precipitation was 0.4 mm. Ambient conditions were obtained from ARSO (Slovenian Environmental Agency; local meteorological station: Ljubljana Bežigrad). After completion of the acute bout of exercise, the dogs underwent a passive 30-minute recovery period during which they remained at rest or in the sternal recumbency.

The study used data collected as part of routine examinations. Written informed consent was obtained from the owners before the dogs entered the study. All procedures complied with relevant Slovenian government regulations (Animal Protection Act, Official Gazette of the Republic of Slovenia, 43/2007).

### Measuring Body Temperature

In each dog, rectal temperature was measured with a digital thermometer (Microlife AG, Widnau, Switzerland) at the same time points as blood sampling, i.e., before and immediately (within 3 min of exercise cessation) after the first acute bout of exercise on day 1, after the second acute bout of exercise on day 2 and after 24 h of rest (day 3). Rectal temperature was measured before blood sampling.

### Blood Sample Collection and Processing

Blood samples for the determination of hematological, biochemical and oxidative stress parameters were taken by venipuncture of the saphenous vein four times: before and immediately (within 3 min of exercise cessation) after the first acute bout of exercise on day 1, after the second acute bout of exercise on day 2 and after 24 h of rest (day 3).

Tubes containing the anticoagulant ethylenediaminetetraacetic acid tri-potassium salt (K3EDTA) (Vacuette; Greiner Bio-One, Kremsmunster, Austria) were used to collect blood samples for the determination of hematological parameters (complete blood count and white blood cell differential count) and MDA concentrations. Hematological analyzes were performed within 4 h of blood collection. Blood samples for determination of MDA concentration were centrifuged at 1,500 × g for 10 min at 4°C. Plasma was collected and immediately stored at −80°C until analysis. Blood samples for the determination of the activities of GPX in whole blood lysates and SOD in washed erythrocytes (erythrocyte SOD1 activity–cytosolic Cu-ZnSOD, which is not tissue specific) were collected in tubes containing lithium heparin (Vacuette; Greiner Bio-One, Kremsmunster, Austria). Samples of whole blood were aliquoted and immediately stored at −80°C until analysis. Lysates of washed erythrocytes were prepared according to the instructions of the reagent kit for the determination of erythrocyte SOD1 activity (Randox, Crumlin, UK), and stored at −80°C until analysis.

Blood samples for the determination of biochemical parameters, with the exception of glucose, were collected in serum separator tubes (Vacuette; Greiner Bio-One, Kremsmunster, Austria) and centrifuged at 1,300 x g at room temperature for 10 min to separate the serum. Biochemical analyzes, including glucose, were performed on the day of blood collection. Blood samples for the determination of glucose concentration were collected in tubes containing sodium fluoride and potassium oxalate (Vacuette; Greiner Bio-One, Kremsmunster, Austria) and centrifuged at 1,500 x g for 15 min at 4°C).

### Blood Analysis

Hematological analyzes were performed with an automated laser-based hematology analyzer (ADVIA 120; Siemens Munich, Germany). With the exception of electrolytes (sodium, potassium, chloride), all biochemical parameters [glucose, urea, creatinine, total protein, albumin, cholesterol, triglycerides, calcium, inorganic phosphate, alanine aminotransferase, alkaline phosphatase, aspartate aminotransferase (AST), and creatine kinase (CK)] were determined using an automated biochemical analyzer (RX-Daytona; Randox, Crumlin, UK). Electrolyte concentrations were measured using an electrolyte analyzer (ILyte; Instrumentation Laboratory, Lexington, MA, USA). Individual whole blood lactate measurements were performed immediately after blood collection using a portable (handheld) dry chemistry instrument (Accutrend, Roche Diagnostics, Mannheim, Germany).

The activities of whole blood GPX and erythrocyte SOD1 were measured spectrophotometrically with an automated biochemical analyzer using commercially available reagent kits [Ransel (whole blood GPX) and Ransod (erythrocyte SOD1); both Randox, Crumlin, UK]. The activities of whole blood GPX and erythrocyte SOD1 were expressed as units per gram of hemoglobin (U/g Hgb). Hemoglobin concentration in red blood cell hemolysates was determined by a cyano-methemoglobin method using an RX-Daytona automated biochemical analyzer (Randox, Crumlin, UK).

Total plasma MDA concentration was determined by alkaline saponification using the derivatization method (67). MDA was derivatized with 2,4-dinitrophenylhydrazine to a pyrazole derivative and determined using an Agilent 1200 series high performance liquid chromatography system (Agilent, Waldbronn, Germany). The derivatized samples were separated on an Agilent Eclipse XBD-C18 column by gradient elution with acetonitrile, water, and acetic acid, and the MDA derivative was detected with the diode array detector at 310 nm. The plasma MDA concentration was expressed as μmol per L (μmol/L).

### Statistical Analysis

Data were analyzed using IBM SPSS (version 25, IBM Corp., Armonk, NY, USA). Descriptive statistics were used to obtain basic information about the variables measured. Due to the small number of dogs included in the study, a non-parametric test was used to assess differences in measured parameters between different sampling times (Friedman analysis followed by multiple comparisons and Bonferroni correction). Results were expressed as medians and interquartile ranges (IQR; 25th to 75th percentiles). A value of *p* < 0.05 was considered significant.

## Results

Seven healthy, client-owned, trained canicross dogs (three females/four males) aged from 12 to 120 months (median age 19 months) with a median weight of 22.8 kg (IQR: 16.4–34.0 kg) were included in the study. Canicross dogs included 3 mixed breed dogs, 1 Greysther (Greyhound/German Short-haired Pointer hybrid breed type), 1 Siberian Husky, 1 Whippet and 1 American Staffordshire Terrier.

All seven dogs completed the two acute bouts of exercise conducted during the two consecutive days. The increase in rectal temperature in participating dogs ranged from 0.1 to 3.0°C. The highest rectal temperature (Table 1) was measured immediately after exercise on day 2 (41.7°C; median value: 41.1°C) and was significantly higher compared to rectal temperature measured before exercise on day 1 (median value: 38.7°C) and after 24 h of rest on day 3 (median value: 38.3°C). After 24 h of rest, rectal temperature was significantly lower compared to rectal temperature measured after exercise on day 1 and day 2, but there was no significant difference when compared to the pre-exercise measurement on day 1.

**Table 1:** Rectal temperature [RT; median, IQR (25th−75th percentile), MIN–MAX (minimum to maximum values)] measured at different blood sampling times in dogs during canicross training session.

	**Pre-exerciseday 1**	**Post-exerciseday 1**	**Post-exerciseday 2**	**After resting day 3**
RT (°C)
Median	38.7	39.1	41.1^b^	38.3^a^
IQR	38.6–38.8	38.9–39.4	40.1–41.3	38.3–38.5
MIN–MAX	38.5–38.9	38.7–39.5	39.4–41.7	38.2–38.6

a *Significant difference (p < 0.05) compared to post-exercise measurements on days 1 and 2*.

b *Significant difference (p < 0.05) compared to before exercise measurements on day 1 and after 24 h of rest (Only day 3)*.

Hematological parameters (Table 2) did not differ significantly between sampling times. On the other hand, some biochemical parameters (Table 3), including urea, creatinine, CK, and triglycerides, changed significantly during the two-day exercise session. Regardless of significant change, the majority of median values of hematological and biochemical parameters remained within their reference ranges at all sampling times. The hemoglobin concentration was slightly above the upper value of the reference range at all sampling times except before exercise on day 1. The median values of albumin concentrations slightly exceeded the upper value of the reference range at all sampling times and the median value of chloride concentration exceeded the upper value of the reference range after exercise on day 2.

**Table 2:** Hematological parameters [median, IQR (25th−75th percentile)] in dogs during canicross training session.

**Measured parameters**	**Pre-exercise day 1**	**Post-exercise** **day 1**	**Post-exercise day 2**	**After resting day 3**	**REF**
WBC ( ×10^9^/L)					5.2–13.9
Median	8.93	7.77	8.53	8.57	
IQR	6.53–11.4	6.76–9.44	7.04–10.9	6.71–13.4	
RBC ( ×10^12^/L)			.		5.7–8.8
Median	7.35	7.52	7.72	7.38	
IQR	6.58–7.96	6.11–8.35	6.93–8.22	6.94–7.81	
HCT (L/L)					0.37–0.57
Median	0.52	0.56	0.55	0.54	
IQR	0.47–0.58	0.44–0.59	0.50–0.59	0.49–0.57	
Hgb (g/L)					129–184
Median	178	187	191	186	
IQR	165–201	154–200	178–201	165–196	
PLT ( ×10^9^)					143–400
Median	204	207	229	217	
IQR	184–278	142–291	206–257	182–286	
NEUT (%)					42.5–77.3
Median	55.5	57.4	58.7	57.3	
IQR	50.1–63.5	52.0–66.9	49.3–70.8	49.1–63.4	
LYMPH (%)	3				11.8–39.6
Median	1.1	30.1	29.3	29.3	
IQR	28.5–35.4	26.0–33.1	21.9–36.8	21.7–35.1	
N/L ratio					
Median	1.78	1.91	2.01	1.97	
IQR	1.40–2.27	1.57– 2.63	1.35–3.24	1.42–2.99	

*WBC, white blood cell count; RBC, red blood cell count; HCT, hematocrit; Hgb, hemoglobin concentration; PLT, platelet count; NEUT, relative number of neutrophils; LYMPH, relative number of lymphocytes; N/L, the ratio between relative numbers of neutrophils and lymphocytes; REF, reference ranges (Advia 120; Siemens, Munich, Germany)*.

**Table 3:** Biochemical parameters [median, IQR (25th−75th percentile)] in dogs during canicross training session.

**Measured parameters**	**Pre-exercise day 1**	**Post-exercise**	**Post-exercise**	**After resting**	**REF^c^**
		**day 1**	**day 2**	**day 3**	
Lact (mM/L)					1.2–3.1^d^
Median	1.00	1.55	1.70	2.20	
IQR	0.80–1.65	1.23–2.63	0.95–3.00	1.95–2.53	
Urea (mM/L)					2.50–9.60
Median	7.76	9.10^a^	8.06	7.53	
IQR	6.80–9.48	6.44–10.5	7.42–9.12	6.25–8.06	
Crea (μM/L)					44.2–132.6
Median	101.1	108.2^a^	106.9	102.1	
IQR	99.7–107.4	107.0–111.3	104.1–116.3	94.7–108.6	
Glu (mM/L)					3.60 −6.55
Median	6.00	5.80	5.70	4.90	
IQR	5.80–6.50	5.20–6.30	4.20–5.90	4.40–5.40	
TP (g/L)					54.0–71.0
Median	62.3	61.3	60.0	61.3	
IQR	59.8–63.5	58.9–63.1	55.7–63.0	60.0–65.4	
Alb (g/L)					26.0–33.0
Median	36.4	35.1	33.7	35.5	
IQR	34.5–38.8	33.3–37.6	32.7–37.9	33.9–35.8	
Chol (mM/L)					3.50–6.99
Median	5.46	5.40	5.54	5.81	
IQR	3.32–7.01	3.35–6.96	3.70–6.19	3.42–6.44	
Trig (mM/L)					0.2–1.3
Median	1.26	0.89	0.98	0.75^b^	
IQR	0.67–1.77	0.87–1.35	0.79–1.34	0.60–1.03	
Na (mM/L)					141–152
Median	150.8	151.1	150.7	150.8	
IQR	149.2–151.1	150.2–152.9	150.4–152.7	150.1–151.6	
K (mM/L)					4.37–5.35
Median	4.39	4.60	4.58	4.62	
IQR	4.37–4.92	4.55–5.00	4.54–4.86	4.45–5.34	
Cl (mM/L)					105–115
Median	114.4	114.6	117.3	114.2	
IQR	112.8–114.7	114.2–115.3	116.6–117.4	113.7–114.6	
Ca (mM/l)					2.25–2.83
Median	2.54	2.58	2.49	2.57	
IQR	2.53–2.64	2.53–2.65	2.45–2.52	2.52–2.65	
iP (mM/L)					0.84–2.00
Median	1.35	1.32	1.34	1.29	
IQR	1.29–1.56	1.22–1.49	1.09–1.47	1.14–1.63	
AP (U/L)					20–156
Median	58.9	59.5	59.5	57.2	
IQR	30.0–85.4	33.3–89.0	33.0–88.4	28.8–85.6	
ALT (U/L)					21–148
Median	61.2	65.1	68.0	60.8	
IQR	47.6–73.6	45.7–80.9	43.7–73.3	48.9–73.9	
AST (U/L)					23–66
Median	34.0	44.0	38.0	42.0	
IQR	25.0–42.0	42.0–45.0	31.0–51.0	24.0–54.0	
CK (U/L)					0–130
Median	70.0	105.5 ^b^	109.5 ^b^	91.5	
IQR	59.3–76.8	71.0–136.0	67.0–139.8	73.0–138.0	

a *Significant difference (p < 0.05) compared to resting values on day 3*.

b *Significant difference (p < 0.05) compared to pre-exercise values on day 1*.

c *Own reference ranges of a diagnostic laboratory, except for lactate concentration*.

d *Stevenson et al. (68)*.

*Na, sodium; K, potassium; Cl, chloride; Ca, calcium; iP, inorganic phosphate; AP, alkaline phosphatase; ALT, alanine aminotransferase; AST, aspartate aminotransferase; CK, creatine kinase; REF, reference ranges; Lact, lactate; Crea, creatinine; Glu, glucose; TP, total protein; Alb, albumin; Chol, cholesterol; Tri, triglycerides; mM/L, mmol/L; μM/L, μmol/L*.

The concentration of urea (Table 3) increased after exercise on day 1 and decreased after 24 h of rest, resulting in a significantly higher (*p* = 0.043) concentration of urea immediately after exercise on day 1 compared to its concentration after rest. A similar change was noted for creatinine concentration, which was significantly higher (*p* = 0.011) immediately after exercise on day 1 than its concentration measured after 24 h of rest. Triglyceride concentration decreased during the 2 days of canicross exercise session, resulting in a significantly lower (*p* = 0.043) triglyceride concentration at the last blood sampling compared to its concentration before exercise on day 1. The activity of serum muscle enzyme, CK, was significantly increased after exercise on days 1 (*p* = 0.022) and 2 (*p* = 0.044) compared to pre-exercise activity on day 1. After 24 h of rest, CK activity decreased to near pre-exercise activity. Blood glucose concentration decreased, and blood lactate concentration increased from the first (pre-exercise, day 1) to last (after a 24-h of rest period day 3) blood sampling; however were not significantly different.

Oxidative stress parameters (Table 4) did not differ significantly between any sampling times.

**Table 4:** Oxidative stress parameters [median, IQR (25th−75th percentile)] in dogs during canicross training session.

**Measured parameters**	**Pre-exercise day 1**	**Post-exercise**	**Post-exercise**	**After resting**
		**day 1**	**day 2**	**day 3**
**SOD1 (U/g Hgb)**				
Median	2,560	2,474	2,563	2,645
IQR	2,135–2,787	2,148–2,780	2,381–2,670	2,240–2,715
**GPX (U/g Hgb)**				
Median	494.0	490.8	478.4	488.4
IQR	441.9–498.2	469.7–506.7	436.9–493.1	446.1–499.9
**MDA (μmol/L)**				
Median	6.54	6.11	6.31	6.50
IQR	5.75 −7.28	5.59–6.68	5.41–6.61	6.04–7.25

*SOD, erythrocyte superoxide dismutase1; GPX, glutathione peroxidase; MDA malondialdehyde; U/g Hgb, units per gram of hemoglobin*.

## Discussion

To the best of the authors' knowledge, no data on hematological, biochemical and oxidative stress parameters in trained canicross dogs have been reported in the literature. Therefore, determining the changes in rectal temperature and hematological, biochemical and oxidative stress parameters before, during and after the two acute bouts of exercise provides valuable information about the health status and physiological responses of canicross dogs to the exertional demands to which these dogs were exposed during their regular two-day exercise sessions. Canicross racing is not directly comparable to Greyhound and sled-dog racing. There are several important differences affecting the physiological responses of canine athletes to these forms of physical activity, which include different demands that sports place upon the canine athletes, duration and the distance of the races, environmental conditions at which different races take place and the training conditions, including human athletes, as well as the breeds of canine athletes. Pre-exercise (day 1) and post-resting (day 3) values of hematological, biochemical and oxidative stress parameters are indicative of training status while post-exercise values reflect the acute stress of the exercise bout within this trained cohort.

### Physiological Parameters

Physical exercise not only leads to oxidative stress, but can also alter physiological metabolism in humans and animals, resulting in significant changes in physiological, hematological, and biochemical parameters. Most of these changes are considered normal responses to physical activity and depend on the duration and intensity of physical activity, as well as the fitness and training level of the athlete (11, 23, 29, 30, 36, 39, 69–71).

The combination of exercise and limited evaporative cooling through panting leads to several physiological changes that include tachypnea, lactic acidosis, respiratory alkalosis, hyperthermia, increased heart rate and hypocapnia (28, 34, 72), dry mucous membranes, prolonged capillary refill time, ataxia, and increased body temperature (73). Exertional heat-related illness (8, 10, 74) occurs when metabolic heat accumulates due to exercise and is a well-recognized threat for canine athletes, leading to severe organ failure and death (8, 10, 75), this is however dependent on maximal rectal temperature as well as to its duration. Physical fitness, heat acclimation and the rapid heat shock response are directly invoked to combat heat related illness (10). Clinical signs of heat-related illness include extended wide and flat tongue, partially or fully closed eyes, retracted ears, seeking shade, and panting (76).

Although unfit healthy dogs have significantly reduced exercise endurance and an increased rate of temperature rise compared to normally active healthy dogs (25), even fit and trained dogs have been shown to have core body temperatures of 38.9 to 42.4°C after exercise (76) and ear temperature of 42.5°C measured post canicross exercise (7). Core body temperature is in close agreement with rectal temperature, but is slightly (0.4°C) higher (7, 77). As invasive measurements of core body temperature can only be performed in some clinical settings (78), it is therefore considered standard practice to measure rectal body temperature using a digital thermometer. The ability to collect this is not tolerated by all dogs (79) and may be impractical in non-clinical situations (80).

Clinically, a core body temperature above 41°C has been historically defined as heat stroke (7, 76, 81) but the certainty of diagnosing the heat-related illness by the temperature value alone is questioned and should therefore be avoided (82). In our study, we observed that even in dogs running in the thermoneutral zone with temperatures below 20°C, rectal temperature can rise up to 41.7°C (Table 1). The increase in rectal temperature in participating dogs ranged from 0.1 to 3.0°C, which is similar to results reported by McNicholl and colleagues (83) and Carter and Hall (7) where the mean increase in body temperature was 2.1°C (rectal temperature) and 1.8°C (tympanic membrane temperature), respectively, and was significantly related to ambient temperature. Similarly, as in the study by Carter and Hall (7), all dogs in our study returned to a normal body temperature soon after the cessation of exercise, which suggests that appropriate cooling mechanisms preventing prolonged hyperthermia are likely in place in these dogs. The participating dogs in this study showed signs of hyperthermia, but despite very high temperatures (maximal measured rectal temperature was 41.7°C), no dogs experienced clinical manifestations of exertional heat-related illness requiring medical intervention. A significant increase in post-exercise body temperature has been also reported in human athletes (18, 19, 84, 85).

Along with heart rate and rectal temperature, blood lactate concentration is one of the most commonly measured physiological parameters for assessing the effort required by exercise in humans and dogs. Blood lactate concentration is used as an indicator of glycolytic activity in skeletal muscle; the degree of increase is related to exercise intensity, fitness, and degree of training as it reflects the dependence on anaerobic metabolic pathways (20, 21, 36, 70, 86, 87).

In our study, the median blood lactate concentration measured before exercise on day 1 was slightly below the lower value of the reference interval for dogs (1.2–3.1 mmol/L) determined with an Accutrend portable lactate meter (68); the same lactate meter was used in our study. Lactate concentration increased gradually, but not significantly, from the second (after the acute bout of exercise on day 1) to the last (after 24 h of rest) blood sampling without exceeding the upper value of the reference interval (68). The lack of blood lactate response to two acute bouts of exercise in the canicross dogs is likely a result of the regular training and the fact that exercise intensity was not sufficiently high to elicit appreciable changes in blood lactate concentrations following exercise. The results of the lactate concentrations measured during the study indicate that the canicross dogs included in this study were in good physical condition. Similar results were observed in search and rescue dogs after a 4-h search and rescue field exercise with handlers (64). Significantly elevated lactate concentrations after exercise were measured in other canine athletes (22–24, 28, 29, 34, 39).

### Hematological Parameters

In our study, all hematological parameters were within the reference intervals for dogs before exercise on day 1. While the median values of hemoglobin concentration were slightly above the upper value of the reference range at all sampling times, except before exercise on day 1, all other hematological parameters remained within the reference ranges at all sampling times. In our study, none of the hematological parameters changed significantly at any sampling time point. Similar to our results, no significant increase in erythrocyte variables was observed in sled dogs after short-term high-intensity exercise (32), in search and rescue dogs after exercise (30, 64), and in trained Labrador retrievers during field trial training and competition (28). The lack of a significant increase in erythrocyte variables in our dogs could be due to the effects of 4 months of training and/or the inconvenient timing of sample collection. Blood collection occurred within 3 min of training, which means we may miss the transient change that is most pronounced in the first minute after the cessation of exercise (32, 88). Dogs and horses are capable of splenic contraction during exercise as a mechanism of increasing the number of circulating red blood cells (RBCs) and the oxygen-carrying capacity of the blood (29, 36, 71, 89). Thus, in dogs, increases in RBC, hematocrit, and hemoglobin concentration are most commonly attributed to mobilization of RBCs from the spleen by sympathetic-induced splenic contraction, as well as exercise-induced fluid shifts, including secondary hemoconcentration due to dehydration, and exercise training (29, 34, 36, 69, 71, 88). Therefore, splenic contraction cannot be completely ruled out in our study as it may occur prior to the collection of the first blood sample as a result of an anticipatory response to exercise. Blood samples were collected immediately prior to the first acute bout of exercise after arrival at the training field, so emotional stress and therefore sympathetic effects cannot be ruled out (32).

### Biochemical Parameters

Apart from median albumin and chloride concentrations, all other biochemical parameters remained within their reference intervals at all sampling time points. The hyperalbuminemia that occurred throughout the study was a surprising finding and could be due in part to the shift of fluids into skeletal muscle cells (32) or most likely to the effect of regular exercise training. In humans, intense exercise has been shown to stimulate albumin synthesis (90, 91). In canine athletes, respiratory alkalosis and metabolic acidosis may occur due to hyperventilation in response to strenuous anaerobic physical activity and high body temperature (28, 64, 72). Electrolyte imbalance may be determined by measuring concentrations of sodium, potassium, chloride and calcium. Slightly elevated or normal chloride concentrations with concomitant decreased sodium and potassium concentrations are indicative of acute respiratory alkalosis (28). In our study, sodium concentrations were close to the upper value of the reference intervals, suggesting that the hyperchloremia observed in our dogs after exercise on day 2 may be due to mild dehydration and intercompartmental fluid shift rather than respiratory alkalosis.

Acute bouts of exercise resulted in significant changes in urea, creatinine, CK, and triglycerides between specific measurement time points. Despite significant changes, these biochemical parameters remained within their reference intervals and are therefore of limited clinical relevance.

As expected, exercise resulted in a slight but significant increase in the activity of CK on days 1 and 2 compared with pre-exercise activity. After a 24-h rest period, the activity of CK decreased. These results suggest transient changes in muscle cell permeability rather than permanent damage to muscle fibers. Similar results were obtained in Labrador retrievers after strenuous exercise (27) and in search and rescue-trained dogs after 20 min of exercise (30). Creatine kinase and AST are most commonly used markers for assessing skeletal muscle damage during exercise in human (14) and canine athletes (36, 92, 93). In dogs the peak value in plasma CK does not provide a realistic assessment of the amount of muscle damaged, making CK a relatively crude indicator of muscle damage (93). Substantially higher exercise-induced increases in CK activity attributable to muscle damage from extremely strenuous exercise have been reported in greyhounds (23), sled dogs (26, 33, 38, 40, 58–61, 88, 94), search and rescue dogs (64), and trained, untrained, and sedentary healthy beagle dogs (95).

Hematocrit, total protein, and creatinine concentrations are common markers of hydration status (hemoconcentration) and fluid shift in human athletes (20) and exercising dogs (36). Assuming an unchanged glomerular filtration rate in canicross dogs, significantly increased urea and creatinine concentrations observed after exercise on day 1 without a significant increase in total protein concentration and hematocrit are likely a result of increased muscle catabolism during exercise (26, 30).

Triglycerides stored in adipose tissue and muscle fibers are considered the main source of free fatty acids oxidized during exercise. Other triglyceride pools include lipoproteins and deposits interspersed between skeletal muscle fibers (96, 97). In exercising dogs, several studies reported an increased concentration of triglycerides after exercise (29, 30, 35, 60), which is in contrast to our findings. During aerobic physical activity, circulating triglycerides are the main source of fat utilized by type I fibers as these contain very high levels of low-lipoprotein lipase (96–98). The latter could be the explanation for the gradual and significant decrease in serum triglyceride concentration during exercise training for two consecutive days.

### Oxidative Stress Parameters

While regular, non-exhaustive physical activity is beneficial to health by improving antioxidant defenses, strenuous and prolonged exercise and training lead to overproduction of ROS and consequently oxidative stress-induced tissue damage and impaired muscle contractility (42–44, 47, 49, 51, 99). Several studies in humans and animals have reported elevated levels of biomarkers of oxidative damage in blood and skeletal muscle as a result of strenuous and/or prolonged exercise (43, 50, 51, 100). Increased levels of markers of oxidative damage and decreased levels of antioxidants have also been reported in canine athletes subjected to various types of exercise (34, 57–61, 63, 101), but not in canicross dogs, making it difficult to compare our results with those of other studies conducted in exercising dogs. These studies differ significantly not only in the type of exercise, breeds included in studies, but also in the types and methods of oxidative stress parameters measured.

The antioxidant enzymes GPX and erythrocyte SOD1, as well as the lipid peroxidation marker MDA determined in our study, are commonly used markers of oxidative stress (102, 103). Glutathione peroxidase and SOD are important antioxidant enzymes that represent the first line of antioxidant defense (104, 105). In canicross dogs prior to the first acute bout of exercise, GPX activity was consistent with our previously reported results (106) obtained in healthy dogs using the same method for GPX determination as in the present study. In contrast, the median erythrocyte SOD1 activity before the first acute bout of exercise was much higher than in our previously reported results (106) obtained in healthy dogs: 2,560 U/g Hgb (IQR: 2,135–2,787 U/g Hgb) and 1,790.8 U/g Hgb (IQR: 1,686–1,999 U/g Hgb), respectively, although the same method was used to determine erythrocyte SOD1 activity in both studies. The high erythrocyte SOD1 activity measured in our study may be due to antioxidant adaptation to training (49–51, 107–109), as the dogs included in the study had trained for 4 months prior to the start of the study. Increased erythrocyte SOD1 activity and decreased MDA concentration was reported in a group of human subjects having regular football training compared to students with sedentary lifestyle (110). Reactive oxygen species produced during exercise act as signals to increase the expression of antioxidant enzymes important for muscle cell adaptation to exercise. Superoxide dismutase levels are commonly higher in resting blood and skeletal muscle of trained individuals than in control groups (44, 47, 49, 51). In addition, it has been shown that intense exercise and regular endurance training led to upregulation of the copper-zinc SOD (erythrocyte SOD1–Cu-ZnSOD; located in the cytosol and mitochondrial membrane; isoform measured in our study) and the tetrameric manganese SOD (MnSOD; located in the mitochondria) in skeletal muscle (46). After acute bouts of exercise, GPX and erythrocyte SOD1 activities did not change significantly in the canicross dogs, which may be due to the current speed of 15 km/h (average), which was not of sufficient intensity for our dogs to trigger sufficient ROS production that would lead to significant changes in GPX and/or erythrocyte SOD1 activities. Another explanation for the lack of significant changes could be the measurement of GPX and SOD1 activities in whole blood and erythrocytes, respectively. We might obtain different results if we could measure skeletal muscle-specific activity of GPX and SOD, but it was not possible to perform muscle biopsy in our canicross dogs. Finally, the lack of significant changes could be due to training-induced hormesis in the antioxidant system because the dogs in our study were regularly trained canicross dogs. There is growing evidence that in humans and animals, antioxidant systems, including enzymatic and non-enzymatic antioxidants, are capable of great adaptation to acute and chronic exercise (44, 46, 49, 51, 111). Similarly to our results, erythrocyte SOD1 and GPX activities did not change significantly in trained sled dogs that completed a 58-km run on each of three consecutive days (58). In another study, Hinchcliff and colleagues reported a significant decrease in erythrocyte activity of GPX and unchanged erythrocyte SOD1 activity in sled dogs participating in a long-distance sled-dog race (59). A significant decrease in erythrocyte SOD1 activity was observed in sled dogs after an 11-day race (101).

Reactive oxygen species oxidize important biological macromolecules such as lipids, proteins, and DNA, thus causing structural and functional changes to these molecules. Lipid peroxidation as a marker of oxidative stress is usually assessed by measuring the concentrations of lipid peroxidation products, such as MDA and F2-isoprostanes (43, 103, 112–114). In our study, the MDA concentration was slightly higher than the MDA concentration measured in healthy dogs (unpublished data) and is in agreement with our previous results obtained in brachycephalic dogs (115) using the same method for MDA measurements.

Virtually the same level of MDA concentrations measured at all sampling times clearly suggests that lipid peroxidation was less likely to occur in our canicross dogs during the two-day exercise session. The reason for this could be the training adaptation of the antioxidant system of our dogs. Trained humans and animals have been found to have lower levels of oxidative damage, such as lipid peroxidation, than their sedentary counterparts (49, 51, 108–110). Despite the differences in the type of physical activity, no significant increase in lipid peroxidation biomarkers was observed after exercise in trained foxhound dogs (35) and sled dogs exercising for two consecutive days (62). On the other hand, increased levels of lipid peroxidation biomarkers have been observed in trained dogs after exercise, e.g. in sled dogs after repeated endurance exercise (58) and in dogs after agility exercise (34).

## Limitations

The present study has some limitations. The first is the small number of dogs included in our study. Our results demonstrate that well-trained, fit, largely experienced canicross dogs do not experience severe effects when they are subjected to two acute bouts of exercise at the reported ambient temperatures. However, this cannot be extrapolated to unfit, less experienced dogs completing the same distance or same ambient conditions. The timing of the study (April) after months of training is important to be considered. The results could have been significantly different if the same study had been conducted in September/October after a period of summer rest, when the dogs may have been less conditioned/acclimatized to exercise. The second limitation is the fact that we were not able to collect blood samples before the start of the training season, which would help us to better interpret our results. The next limitation is the timing of sample collection prior to the first exercise session. The latter was performed immediately prior to the first acute bout of exercise after arrival at the training field so our pre-exercise results likely reflect an anticipatory response. Excitability, apprehension, and anticipation of exercise at the time of blood sampling may lead to catecholamine-mediated splenic contraction, which is reflected in changes in hematological and biochemical parameters. Therefore, blood samples for evaluation of pre-exercise blood values (resting, baseline) should be collected temporally distant from the exercise event; ideally several hours prior to exercise when dogs are in a home environment with their owners. Transport can also alter hematological and biochemical results (28, 30, 32, 71). The final limitation of our study could be the difference in fitness level of the canicross dogs and their owners. However, except for one dog, this was not their first season of racing. Canicross is a sport that connects canine and human athletes in running. Therefore, the dogs' response to exercise is limited by the fitness level of the human athlete. In our study, all dogs and their owners were well-trained athletes participating in canicross competitions, but they were not professional athletes.

## Conclusions

The present study provides valuable information on the health status assessed by measuring the rectal temperature, hematological, biochemical and oxidative stress parameters of trained canicross dogs before and during their regular two-day exercise training session. Based on our results, we can conclude that the trained canicross dogs included in our study were healthy, in good physical condition, and fit for the two acute bouts of exercise conducted during two consecutive days. The relatively high erythrocyte SOD1 activity, measured at all measurement time points, may indicate antioxidant adaptation to regular exercise training. However, no significant differences in oxidative stress parameters were found between any sampling times. Exercise resulted in a significant increase in rectal temperature and slight but significant changes in some biochemical parameters, indicating a normal physiological response of canicross dogs to two acute bouts of exercise.

Finally, our results warrant further studies in a larger group of canicross dogs and the measurement of other markers of oxidative stress as well as hematological and biochemical parameters and extracellular vesicles, simultaneously in canicross dogs and their owners. Further research on the effects of canine fitness, ambient temperature, and duration of exercise (e.g., with less fit, slower human runners) is also needed.

## Data Availability Statement

The raw data supporting the conclusions of this article will be made available by the authors, without undue reservation.

## Ethics Statement

Ethical review and approval was not required for the animal study because the study used data collected as part of routine examinations. The request for approval of our research was sent to the Animal Health and Welfare Division of the Administration of the Republic of Slovenia for Food Safety, Veterinary Sector, and Plant Protection. This is the institution in Slovenia responsible for the approval of any study or research conducted on animals. Our application was considered and after finding out that client-owned animals will be subjected to routine clinical exams, including blood collection on four occasions, requested by their owners the Administration concluded that in accordance with the rules on conditions for animal experiments in the proposed study no additional procedures to routine clinical examination would be performed therefore no special approval for procedures on animals is needed. Written informed consent was obtained from the owners before the dogs entered the study. All procedures complied with relevant Slovenian Government Regulations (Animal Protection Act, Official Gazette of the Republic of Slovenia, 43/2007).

## Author Contributions

VE contributed to the conceptualization, data curation, investigation, methodology, and writing–original draft preparation, review, and editing. TV was involved in the funding acquisition, investigation, methodology, data curation, and writing–review and editing. ANS participated in the conceptualization, funding acquisition, investigation, formal analysis, data curation, methodology, supervision, and writing–original draft preparation, review, and editing. All authors contributed to the article and approved the submitted version.

## Funding

This research was funded by the Slovenian Research Agency (ARRS), Grant Nos. P4-0053 and P1-0189. The APC was funded by the Slovenian Research Agency, Grant No. P4-0053. The funders were not involved in the study design, collection, analyses, and interpretation of data, or writing of the manuscript.

## Conflict of Interest

The authors declare that the research was conducted in the absence of any commercial or financial relationships that could be construed as a potential conflict of interest.

## Publisher's Note

All claims expressed in this article are solely those of the authors and do not necessarily represent those of their affiliated organizations, or those of the publisher, the editors and the reviewers. Any product that may be evaluated in this article, or claim that may be made by its manufacturer, is not guaranteed or endorsed by the publisher.
